# Editorial: 2022 in review: allergen immunotherapy

**DOI:** 10.3389/falgy.2024.1431413

**Published:** 2024-05-23

**Authors:** Linda Cox, Mike Kulis

**Affiliations:** ^1^ Medicine and Dermatology, Nova Southeastern University, Fort Lauderdale, FL, United States; ^2^ Pediatric Allergy & Immunology, University of North Carolina School of Medicine, Chapel Hill, NC, United States

**Keywords:** allergen immunotherapy, food allergy, sublingual immunotherapy, oral immunotherapy, epicutaneous immunotherapy, peanut allergy, immune adjuvants

**Editorial on the Research Topic** 2022 in review: allergen immunotherapy

It has been three years since the launch of Frontiers in Allergy. In 2022, I was invited to join the editorial board as the Section Editor of Allergy Immunotherapy (AIT). At the time, the Journal had 5 sections and no impact factor. In the short time since its launch, the journal has grown to have 13 sections. In 2023, the journal had 636 citations of the 236 publications from 2021 to 2022. It is predicted that the journal will see its first impact factor in 2024. The AIT section saw similar growth. The section currently has four research topics. Research topics are generally broad in scope and intended to attract original research, as well as well-written reviews. Topics can range from cutting-edge advances in treatment or diagnosis to unmet needs and burning questions. One of the section's open Research Topics is dust mite allergy. We selected this topic because worldwide, dust mites are the most prevalent aeroallergen (1), and the most widely studied indoor allergen. In addition to respiratory allergy, ingestion of dust mite-contaminated foods has been implicated in idiopathic anaphylaxis (2, 3), eosinophilic gastroenteritis (4–6), and possibly ulcerative colitis (4). Whether allergen immunotherapy can effectively prevent or cure these conditions is yet to be determined (6). Additionally, there are dust mite species under investigation and in clinical use that clinicians may not be aware of such as *Dermatophagoides Siboney* (7). *Dermatophagoides Siboney* was initially thought to be limited to the subtropical and tropical climates in South America (8). However, in the past decade, it has been identified as a causative allergen in other regions such as China (9, 10).

The five articles published in Allergy Immunotherapy Year in Review research topic reflect the diversity of the studies on AIT published in 2022 per PubMed. One study compared a novel formulation and delivery method with an aluminum hydroxide adjuvant vaccine (Warmenhoven et al.). Aluminum hydroxide has been used as an adjuvant in AIT for decades. Although it is widely accepted as a safe vaccine adjuvant, there are concerns about the possible toxicity of long-lasting repeated exposure to aluminum during SCIT. In a murine model, investigators compared a cationic allergen-bearing liposome anchored to Bet v1 with an alum-adsorbed Bet v 1 (Warmenhoven et al.). The liposome was created by coiled-coil formation which formed a peptide pair that anchored Bet v 1 to the surface of cationic liposomes surface. The coiled-coil liposome conjugated to Bet v 1 demonstrated lower allergenicity (15-fold) and greater immunogenicity than the soluble Bet v 1 and alum-adsorbed Bet v 1, respectively. The humoral response was accompanied by a strong IL-10 induction upon stimulation with Bet v 1. The authors concluded the cationic liposome's hypo- allergenicity combined with their strong immunogenicity made them a promising alternative for aluminum salt-adsorption allergen extracts.

The 4 other articles on the research topic addressed food allergy (FA). The incidence of IgE-mediated food allergy (FA) has continued to increase over the years which places a substantial burden on patient health and quality of life. With no cure for this disease, the mainstay of management has been allergen avoidance. Fowler and Lieberman provide an overview of the evolving food immunotherapy market (Fowler and Lieberman). Recent advancements in FA treatment have included novel delivery systems and combination treatments, e.g., biologics, probiotics, probiotics, biologic agents, Vitamin D, and Chinese herbs, etc. In 2020. A landmark event occurred: The FDA approved the first product for FA, PALFORZI (Aimmune Therapeutics, Inc.), a peanut powder administered orally.

Johnson-Weaver provides a review focused on the unmet needs of oral immunotherapy **(**OIT) (Johnson-Weaver). These include the requirement for daily dosing and the risks of severe adverse events. Preclinical research studies in animals are investigating different approaches that include lower allergen doses (i.e., below the individual's activation threshold) given on a daily or less frequent basis and alternative routes. The alternative routes that have been explored in animal models include sublingual, epicutaneous (EPIIT), and intranasal immunotherapy.

Herve et al. provide an up-to-date overview of the status of EPIT (Herve et al.). The advantage of EPIT is that it targets the highly immunocompetent, non-vascularized epidermis, which reduces the risk of allergen passage into the bloodstream. Multiple approaches to EPIT are under investigation primarily for the treatment of food allergy. Efficacy has been demonstrated with aeroallergens as well, but FA is the current focus of most investigations. The approaches under investigation include allergen-coated microneedles, application of allergen on the pretreated skin with various methods: tape stripping, abrasion or laser-mediated micro-perforation, and application of allergen to intact skin using an occlusive epicutaneous system.

The article goes on to focus on a specific delivery system the Viaskin technology platform. Viaskin is an occlusive patch containing dried native allergen extracts, without adjuvants. Treatment involved frequent application of small amounts of allergen to the epidermis through occlusion of the intact skin. Preclinical studies have demonstrated that Viaskin can induce potent and long-lasting T-regulatory cells. Most of the clinical trials have focused on peanut allergy and young children. The advantage of this treatment approach is that it is a non-invasive therapy that does not restrict daily activities. However, in 2020, the FDA did not approve the Viaskin patch due to concern the patch wouldn't be as effective if not fully adhered to the skin, and more clinical trials were requested (11).

In 2023, the FDA confirmed that the Phase 3 study met the primary endpoint, but additional safety data would be needed to support a Biologics License Application (12).

One of the challenges with food AIT is determining who can discontinue immunotherapy and what duration is needed to achieve sustained unresponsiveness. Rambo et al. attempt to address this question (Rambo et al.). The study assesses the immunologic responses of twenty peanut-allergic participants enrolled in a phase 3 Palforzia before and after treatment.

Pre- and post-trial sera collected 12 months apart were tested for IgE and IgG4 binding to intact peanut proteins. The study found statistically significant decreases in IgE binding for intact Ara h 2, 3, and 6. Newly identified IgE epitopes were shown to exhibit shifts towards IgG4 binding post-OIT. Palforzia altered the IgE and IgG4 binding ratios and intensity.

The authors concluded that the increase in IgG4 was more important than the decrease in IgE. This study provides some useful knowledge of IgE and IgG4-binding allergen patterns and peptide biomarkers that may help predict desensitization or sustained unresponsiveness.

Moving forward into the last year of the first quarter of this century, I anticipate we will continue to see studies exploring novel delivery systems for both airborne and food allergens. Hopefully, we will see these novel products in Phase II and III clinical trials, as this is a necessary step for regulatory authority approval. The delivery route that is generating a lot of attention and study is EPIT. I predict that there will be a “deeper” investigation of this route (pun is intended) with microneedle technology. It will be important that after the safety and efficacy of a novel delivery system or formulations are established that there be adherence and pharmacoeconomic evaluations because even if a product is extremely effective and safe, if it is cost-prohibitive or cumbersome to use, it is not likely that it will be “used” at all.
